# Emerging Roles of Micrornas in Veterinary Cardiology

**DOI:** 10.3390/vetsci9100533

**Published:** 2022-09-28

**Authors:** Ana Reis-Ferreira, Joana Neto-Mendes, Carmen Brás-Silva, Luís Lobo, Ana Patrícia Fontes-Sousa

**Affiliations:** 1 Hospital Veterinário do Porto, Travessa Silva Porto 174, 4250-475 Porto, Portugal; 2 ICBAS-UP, Instituto de Ciências Biomédicas Abel Salazar, Universidade do Porto, Rua Jorge de Viterbo Ferreira 228, 4050-313 Porto, Portugal; 3 UnIC@RISE, Department of Surgery and Physiology, Faculty of Medicine of the University of Porto, 4200-319 Porto, Portugal; 4 Faculdade de Medicina Veterinária, Universidade Lusófona de Humanidades e Tecnologias, Campo Grande 376, 1749-024 Lisboa, Portugal; 5 Centro de Estudos de Ciência Animal, Campus Agrário de Vairão, 4480-009 Vila do Conde, Portugal; 6 Departamento de Imuno-Fisiologia e Farmacologia, Centro de Investigação Farmacológica e Inovação Medicamentosa (MedInUP), Universidade do Porto, Instituto de Ciências Biomédicas Abel Salazar, Universidade do Porto, Rua Jorge de Viterbo Ferreira 228, 4050-313 Porto, Portugal; 7 UPVET, Hospital Veterinário da Universidade do Porto, Rua Jorge de Viterbo Ferreira 132, 4050-313 Porto, Portugal

**Keywords:** microRNAs, cardiovascular diseases, biomarker, dog, cat

## Abstract

**Simple Summary:**

MicroRNAs are promising novel biomarkers for the diagnosis and prognosis of cardiovascular diseases. These molecules are defined as a class of short-sequence non-coding RNAs that influence the expression of numerous genes. The growing understanding of cardiac biology contributed to recognising specific abnormal microRNA expression when diseases are present, which makes them potential biomarkers and therapeutical targets. Recent studies have analysed and discussed microRNA expression in cardiac diseases, such as myxomatous mitral valve disease, which are prevalent in our animal companions. This review summarises the most relevant microRNAs related to cardiovascular diseases in dogs and cats. In addition, it describes microRNA’s basic biology and function and discusses their potential as circulating biomarkers for diagnosis, prognosis and monitorisation of treatment, as well as their limitations. Although current studies describe microRNA expression in veterinary cardiology, further work is warranted before they are implemented in the clinical setting.

**Abstract:**

Over the last years, the importance of microRNAs (miRNAs) has increasingly been recognised. Each miRNA is a short sequence of non-coding RNA that influences countless genes’ expression and, thereby, contributes to several physiological pathways and diseases. It has been demonstrated that miRNAs participate in the development of many cardiovascular diseases (CVDs). This review synopsises the most recent studies emphasising miRNA’s influence in several CVDs affecting dogs and cats. It provides a concise outline of miRNA’s biology and function, the diagnostic potential of circulating miRNAs as biomarkers, and their role in different CVDs. It also discusses known and future roles for miRNAs as potential clinical biomarkers and therapeutic targets. So, this review gives a comprehensive outline of the most relevant miRNAs related to CVDs in Veterinary Medicine.

## 1. Introduction

For many years, it was believed that the genome’s non-coding regions were not vital as they did not carry information for protein synthesis. However, now it is known that most of the eukaryotic transcriptome is composed of non-coding RNAs [1]. There is a wide variety of non-coding RNA classes, but microRNAs (miRNAs) have been given special attention due to the association of their dysregulation with the development of phenotypic and pathologic alterations [2]. 

MicroRNAs were first discovered in 1993 in the *Caenorhabditis elegans*, a nematode, and since then, they have been identified in viruses, plants, and animals [3]. MicroRNAs are a class of single-stranded endogenous small non-coding RNAs, about 21 nucleotides in length. They are transcriptionally regulated in a manner identical to typical messenger RNA (mRNA) and, when processed, can silence or downregulate the expression of their targets [4]. Most mature miRNAs are processed from long primary transcripts in a stepwise process involving a series of endonucleolytic cleavages. The mature miRNAs are loaded in a large protein complex known as the RNA-induced silencing complex. MicroRNAs guide the RNA-induced silencing complex to complementary target mRNAs, which are translationally repressed or cleaved [5]. 

In the clinical setting, biomarkers can help with the early diagnosis of diseases, evaluate and manage the response to therapy, and assess patient prognosis. MicroRNAs have numerous characteristics that potentially make them suitable new and non-invasive biomarkers [6].

Circulating miRNAs have been detected in peripheral blood and body fluids such as saliva, urine, and breast milk [7]. They can be secreted or produced due to various events like passive leakage from cells and active secretion via cell-derived membrane vesicles or a protein–miRNA complex [8]. There are two circulating miRNA populations; one can be found in proteins like argonaute-2 and high-density lipoproteins. The other is associated with vesicles such as exosomes, microvesicles, and apoptotic bodies [9]. Exosomes are phospholipid bilayer nanovesicles that carry substances such as lipids, proteins, DNA, and non-coding RNA [10]. They can be secreted by almost all eukaryotic cells, and they are a form of cell-to-cell communication in physiological and pathological conditions [11]. Several clinical studies characterised exosomal miRNAs (ex-miRNAs) and validated their potential clinical applications. Still, there is a need for more significant cohorts of patients and standardisation and consistency in the sample processing [12]. These molecules are highly stable and resistant through miRNA binding with carriers that grant protection from degradation. MicroRNAs can outstand conditions such as boiling, acidic or basic pH mediums, and room temperature storage for extended periods [13]. 

MicroRNAs can be quantified in cells, tissues, and biological fluids. Many commercial RNA extraction kits, such as chloroform-phenol-based extraction, magnetic bead extraction, and column-based extraction, are available. Afterward, RNA expression can be analysed using different assays like northern blot, microarray, next-generation sequencing (RNAseq), droplet digital polymerase chain reaction, and real-time polymerase chain reaction (RT-qPCR). Among these methods, RT-qPCR is the gold standard for measuring circulating miRNAs [14].

MicroRNAs regulate various biological processes, including immune response, hematopoietic development and function, tumour suppression, and tumorigenesis [15]. The growing understanding of cardiac biology contributed to recognising specific miRNAs as novel biomarkers for several cardiovascular diseases (CVDs) as they participate in processes such as cardiomyocyte differentiation, growth and contractility, and cardiac rhythm [16]. Abnormal miRNA expression is associated with pathological processes such as congenital defects, arrhythmias, cardiac hypertrophy, and heart failure (HF) [17]. In fact, these molecules play a role in several molecular pathways related to CVDs, such as cardiac fibrosis, through the transforming growth factor-beta and mitogen-activated protein kinase pathways, among others; cardiac hypertrophy, by calcium signalling and cell cycle-related pathways; and angiogenesis, via the vascular endothelial growth factor and other angiogenic pathways [18,19,20]. 

The nomenclature of miRNAs incorporates the prefix “miR-”, except for the “let-” family, which includes some of the first discovered miRNAs. This prefix is then followed by an identifying number that reflects their discovery order. In the case of sequences differing only in one or two nucleotides, an additional lower-case letter is added to the name, for example, “miR-208a” or “miR-208b”. Moreover, the species may be specified using a three- or four-letter prefix, such as “cfa-“ for *Canis familiaris* [21].

## 2. MicroRNAs in the Diagnosis of Cardiovascular Diseases

### 2.1. Heart Development

Deleting critical genes essential to miRNA biogenesis revealed its importance in the cardiovascular system. The expression blockage of all these miRNAs in mice resulted in death during early gestation due to significant development defects of the heart and blood vessels [22]. 

MicroRNA expression profiling studies have stated that only 18 miRNA families account for approximately 90% of cardiac miRNAs [23]. Among cardiomyocytes, miR-1 and miR-133 are the most abundant, both derived from common primary transcripts and promote mesoderm formation from embryonic stem cells [24]. During heart development, the contractile protein expression is strictly regulated by miR-208a, miR-208b, and miR-499, which control myosin gene expression [25]. 

Moreover, it was found that miR-195 is upregulated after birth and contributes to cardiomyocyte mitotic arrest. Precocious overexpression of miR-195 is associated with ventricular septal defects and ventricular hypoplasia [26]. On the other hand, deleting the miR-17~92 cluster leads to severe lung hypoplasia, an evident ventricular septal defect, and consequently to death. This cluster cotranscribes six mature miRNAs: miR-17, miR-18a, miR-19a/b, miR-20a and miR-92 [27].

### 2.2. Arrhythmias

Atrial fibrillation (AF) is one of the most diagnosed tachyarrhythmias in dogs and humans [28,29]. This is the most prevalent arrhythmia in dilated cardiomyopathy (DCM); nevertheless, it can also occur in dogs with myxomatous mitral valve disease (MMVD). Dogs with over 20 kg of body weight and concurrent congestive heart failure (CHF) are more likely to develop AF [30]. Several factors are associated with initiating and maintaining AF, such as structural, electrical, and autonomic nerve remodelling [31].

The miRNA expression level is altered in patients with AF [32]. In atrial fibroblasts, the expression of the extracellular matrix (ECM) genes collagen-1A1 (COL1A1), collagen-3A1 (COL3A1), and fibrillin is modulated by miR-29b. Plasma from human patients with AF and atrial tissue from dogs with AF showed decreased expression of miR-29b. Therefore, miR-29b likely plays a role in atrial fibrotic remodelling and might have value as a biomarker or therapeutic target [33]. Moreover, miR-21 seems to be involved in profibrotic collagen production by regulating COL1A1 indirectly by targeting Sprouty homolog-1 [34]. On the other hand, miR-133 and miR-30 are anti-fibrotic and play a relevant role in the structural alterations observed in chronic AF [35]. 

Qiao et al. stated that miR-132 is downregulated in canine and human patients with AF. The downregulation of miR-132 and the upregulation of the connective tissue growth factor (CTGF), which is an essential player in the process of fibrosis, suggests a molecular mechanism associated with the development of AF-dependent fibrosis. This fact may provide a potential therapeutic target for AF treatment in the future [36]. As already stated, autonomic nerve remodelling is linked to the generation and maintenance of AF [37]. In a canine model of AF, overexpression of miR-206, through the increased production of reactive oxygen species, induced cardiac autonomic nerve remodelling and changes in electrophysiological properties, which facilitate the development of AF [38].

MiR-208 is a relevant cardiac-related miRNA that is associated with cardiovascular health. Previous reports showed that its abnormal expression in mice and humans was associated with arrhythmias [39]. However, a study that included 28 dogs presenting different cardiac rhythm disturbances did not detect any miR-208 plasmatic expression [40].

### 2.3. Myxomatous Mitral Valve Disease

Myxomatous mitral valve disease is dogs’ most common cardiac disease [41]. There is an overproduction of glycosaminoglycans and proteoglycans in diseased valves, disrupting the collagen and elastin fibres in heart valves [42]. Usually, this progressive degeneration occurs in the mitral valve apparatus but does not exclude the involvement of other cardiac valves [41]. MMVD can be staged according to the disease’s severity into four groups, from stage A to D, as recommended by the latest guidelines [43]. Its prevalence increases with age and is more prevalent in small- to medium-sized breed dogs [44].

When evaluating the expression of circulating ex-miRNAs in dogs with MMVD, changes in these miRNAs are detected in dogs as they become older (miR-9, miR-495, and miR-599) and develop MMVD (miR-9 and miR-599) or CHF (miR-181c and miR-495) [45].

It was hypothesised that miRNAs are involved in the development of MMVD and that valvular interstitial cells (VIC) probably endure disease-relevant changes. The expression of miRNAs in VIC from canine mitral valve tissues was analysed using RT-qPCR and RNAseq. Using both methods, let-7c, miR-17, miR-20a, and miR-30d were significantly downregulated in VICs from diseased valves compared to healthy ones, suggesting that they may participate in the development of canine MMVD. This decrease in let-7c, mir-17, and miR-20a is thought to contribute to myofibroblastic differentiation and cell senescence. In contrast, reducing miR-30d may disinhibit cell apoptosis, so these four miRNAs should be further investigated as potential therapeutic targets [46].

A study with microarray technology combining bioinformatics platforms was used to analyse transcript changes in Cavalier King Charles Spaniels (CKCS) with MMVD compared to normal dogs (non-CKCS). They characterised the MMVD transcriptome, identifying several genes associated with inflammation, cell movement, and extracellular matrix organisation. In addition, 59 canine microRNA family members (cfa-mir-RNA) were identified [47]. 

There is evidence that the miRNA expression profile in dogs with MMVD differs between ACVIM stages. Dachshund is one breed prone to develop MMVD, which ultimately causes heart failure (HF). Compared with unaffected dogs, miR-30b is significantly downregulated in ACVIM stage B [48]. This miRNA downregulates CTGF, a profibrotic protein [49], and could potentially be used as a biomarker of ACVIM stage B [48]. Moreover, it appears that expression changes are greater as the disease progresses. Seven miRNAs, cfa-miR-302d, cfa-miR-380, cfa-miR-874, cfa-miR-582, cfa-miR-490, cfa-miR-329b, and cfa-miR-487b appeared to be downregulated whereas four, cfa-miR-103, cfa-miR-98, cfa-let-7b, and cfa-let-7c, were upregulated in stage B1/B2 or C/D, when compared with stage A dogs. When comparing stages B1/B2 and C/D, the expression of cfa-miR-582 and cfa-miR-487b was higher in the latest groups, whereas cfa-miR-103, cfa-miR-98, cfa-let-7b, and cfa-let-7c were downregulated in the stage C/D dogs. This fact suggests that miRNAs might be useful biomarkers for diagnosis, prognosis, or monitoring response to treatment in dogs with MMVD [50].

**Figure 1:**
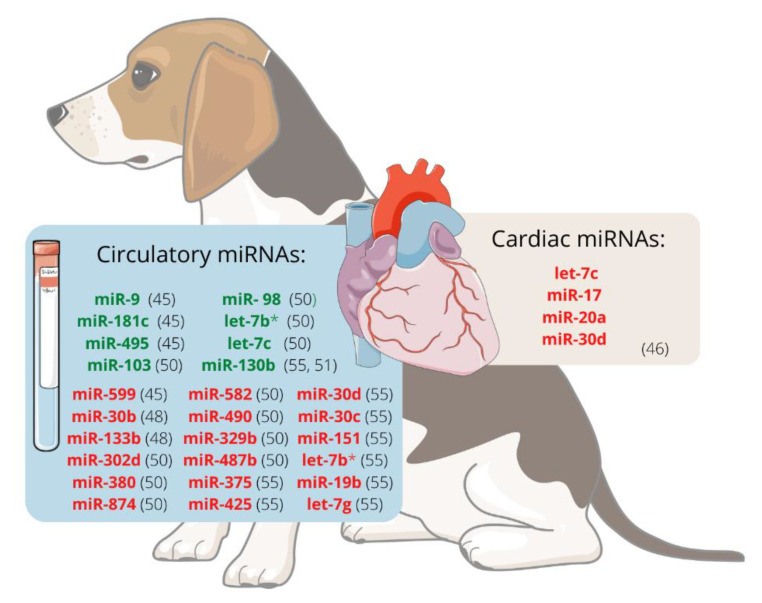
Expression of miRNAs in dogs with myxomatous mitral valve disease. The level of miRNA expression is represented by green if they are upregulated and by red if they are downregulated. let-7b* can be either downregulated or upregulated, depending on the considered study [50,55]. Illustration used elements from Servier Medical (http://smart.servier.com).

Additionally, the expression profile of different miRNAs in dogs with left ventricle eccentric hypertrophy induced by MMVD has already been studied. When stage C and D dogs were compared to healthy controls, one miRNA (cfa-miR-130b) was significantly upregulated and eight were significantly downregulated (cfa-miR-375, cfa-miR-425, cfa-miR-30d, cfa-miR-30c, cfa-miR151, cfa-let-7b, cfa-miR-19b, cfa-let-7g). Furthermore, when the same stage C and D dogs were compared to dogs with cardiac concentric hypertrophy caused by pulmonary stenosis, certain miRNAs appeared to be inherently tied to cardiac hypertrophy. In contrast, others were associated with the underlying disease. In a later study, no statistical difference was found between the expression of cfa-miR-130b in stage C/D dogs and healthy controls. This miRNA was upregulated in dogs with MMVD stage B compared to healthy dogs [51]. Considering the evidence supported by the previous studies, Figure 1 represents the miRNAs differentially expressed, either in plasma/serum or cardiac tissue, in dogs with MMVD.

According to these studies, miRNAs are involved directly or indirectly at the beginning of mitral valve disease and its complications, associated with mechanisms responsible for their severity [52]. MMVD is functionally and histologically similar to mitral valve prolapse (MVP) in humans [42]. There may be ruptured chordae or poor coaptation of the valves due to MVP, which leads to mitral regurgitation (MR) and, eventually, CHF [53]. Recently, Songia et al. evaluated the circulating miRNA profile in human plasma from patients with MVP and observed a strong association between circulating miRNA and MVP pathology. In fact, it was noted that miR-140-3p, 150-5p, 210-3p, 451a, and 487a-3p were significantly upregulated in MVP, and miR-223-3p, 323a-3p, 340-5p, and 361-5p were significantly downregulated, as compared to healthy patients [54]. To the authors’ knowledge, none of these miRNAs was associated with MMVD in dogs. MiRNAs are potential predictive, diagnostic, and prognostic biomarkers for mitral valve diseases and promising therapeutic targets. Still, the molecular pathophysiology of MVP and MMVD needs to be better understood first [45,52].

### 2.4. Cardiomyopathies

After MMVD, DCM is dogs’ second most frequent form of acquired heart disease [56]. This disease is characterised by reduced myocardial contractility, cardiac enlargement, an impaired systolic (and sometimes diastolic) function of one or both ventricles, and possibly arrhythmias. Low cardiac output due to the progressive dilatation of the cardiac chambers will cause weakness and syncope, leading to cardiogenic shock in the end [57]. On the other hand, increased diastolic dysfunction contributes to venous congestion and CHF. It is considered an idiopathic disease as its cause is not yet well understood; nevertheless, it is thought that DCM results from different pathologic processes and has a genetic basis. Usually, medium- to large-sized dogs are the most affected by DCM [56].

A study carried out in 2013 compared miRNA expression patterns between Doberman Pinschers with DCM and healthy ones. They screened serum miRNA expression profiles using miRNA microarray but did not find statistical significance in the 22 miRNAs that appeared to be differently expressed in the two groups. From these 22 miRNAs, five (miR-142-3p, miR-144*, miR-21, let-7c, and miR92a) were selected for further analysis using RT-qPCR, as these were previously mentioned as being involved in cardiovascular pathology. Note that miR-144* and miR-144 are the two strands of the double-stranded precursor miRNA (mir-144). The absence of no statistically significant differences observed in this RT-qPCR analysis may be explained by the small sample size (only four animals per group) [58].

In humans, DCM is the third most common cause of HF and is linked with high morbidity and mortality rates. As in the dog, this disease results from a combination of different pathologic pathways [59]. Left ventricle reverse remodelling (LVRR) occurs with the ideal treatment and improves the left ventricle’s morphology and function. One of the strongest predictors for the development of HF in patients with DCM is LVRR, so Dziewiȩcka et al. tried to relate it with some circulating and tissue microRNAs. From all studied miRNAs, only myocardial miR-133a expression was increased in patients with LVRR; this evidence suggests that miR-133a is involved in cardiac remodelling in DCM [60], which so far has not been confirmed in the dog. 

Nevertheless, at this point, there is no clear benefit in using microRNAs in the clinical setting, as their potential as biomarkers for DCM in veterinary medicine is still quite uncertain. As miR-133, many other microRNAs are relevant in human studies [61]. Further research could help diagnose DCM and risk factors stratification and identification [62].

Hypertrophic cardiomyopathy (HCM) is a spontaneously occurring cardiac disease of the cat. It is described as increased cardiac mass due to left ventricular concentric hypertrophy. There is no obvious aetiology for this disease, but a heritable genetic predisposition is verified in some cases [63]. Also, it is thought that pressure overload (e.g., systemic arterial hypertension) or hormonal stimulation (e.g., hyperthyroidism) influence an HCM phenotype development. This disease usually culminates with the development of CHF due to diastolic dysfunction, which occurs even before chamber remodelling is detected [57]. Cats with primary HCM show a different miRNA profile than healthy ones, including miRNAs that are differentially regulated in humans with cardiovascular diseases. In diseased cats, miR-381-3p, miR-486-3p, miR-4751, miR-476c-3p, miR-5700, miR-513a-3p, miR-320e and miR-1246 appeared to be upregulated [64]. In humans, HCM is a common inherited cardiomyopathy [65]. Epigenetic changes, like the ones carried out by miRNAs, may be involved in the pathogenesis of HCM since only a tiny percentage of patients carry known genetic mutations. Several studies analysed miRNAs’ role in HCM progression in humans, though only a few miRNAs overlap between studies (miR-29, miR-21, miR-133, and miR-1) [61]. Song L. et al. found that miR-1246 and miR-486-3p were downregulated in the cardiac tissue of HCM human patients compared to controls [66]; in contrast, in cats with HCM, upregulation of these miRNAs was observed [64]. The study of HCM may help to disclose pathophysiologic pathways that may be useful for diagnosis, prognosis, and therapeutics of human HCM; therefore, more studies are warranted [67].

### 2.5. Heartworm Disease

Heartworm disease, also called dirofilariasis, is mainly caused by *Dirofilaria immitis*. If not treated, this parasite can cause severe pulmonary and cardiac disease and even death in dogs [68]. In most cases, the adult stages of *D. immitis* are responsible for developing clinical signs. They reside in the pulmonary arteries and, eventually, in the right ventricle, in infections with high worm burdens. Microfilariae, the first larvae stage, are found in circulation as they are produced and released into the bloodstream by adult heartworms. When mosquitos feed on infected animals, these microfilariae are ingested and moulted. The resultant larvae are infective and are transmitted to another animal by the feeding mosquito, making these insects vector for the transmission of this disease [69]. Cats are not usually the preferred target for feeding mosquitos and are inherently more resistant to dirofilariasis, which explains why they are not as frequently infected by heartworms [70].

This disease is more prevalent in the tropics, subtropics, and temperate areas, but predictive models have shown that this disease is spreading to places previously unaffected. The movement of infected dogs and global warming, which both increase the mosquito activity period, is probably why this parasite and vectors are more and more found in regions where they have not been reported before [71].

In 2014, the presence of circulating filarial-derived miRNAs in the host bloodstream was studied by Tritten et al. They detected over 200 mature miRNA sequences that possibly originated from nematodes in the plasma of dogs infected with *D. immitis*. Both miR-34 and miR-71 were found in all samples from *D. immitis*-infected dogs. However, these miRNAs were also detected in samples from dogs infected with *Brugia pahangi* (but never appeared in the plasma of uninfected dogs). Since these two miRNA mature sequences can also be found completely conserved in other nematodes, they may not distinguish the presence of different species. Besides, a low correlation was found between miRNA copy numbers and microfilaria counts, implying that adults also significantly release miRNAs into the bloodstream. This also means that filarial-derived miRNAs can be found in plasma/serum even when the parasite does not exist in the bloodstream [72].

Further studies determined if the intensity of adult worm infection could be distinguished using miRNAs as biomarkers and if they could be used to identify new infections. By measuring plasma levels of miR-34 and miR-71, there was no significant difference in expression between low- and high-intensity infections in dogs infected with *D. immitis* adult worms. When comparing the infected and non-infected groups, there was a substantial difference as the copy number of both these miRNAs was elevated in dogs with *D. immitis*. This reiterates that miR-34 and miR-71 could be used as biomarkers for identifying *D. immitis* infection in dogs, even though their copy number does not reflect the intensity of adults in the host. There is the theory that these miRNAs are stage-specific, as miR-34 is released by both microfilaria and adult worms, while miR-71 only seems to be released by microfilaria [73].

Nevertheless, since these miRNAs are not specific to *D. immitis*, the hypothesis of a co-infection with other nematodes should never be out-ruled [73]. The process by which nematodes release miRNAs into the host bloodstream is still not understood. The central hypotheses are worm death and its disintegration releases miRNAs or that nematodes also release microvesicles (i.e., exosomes) with miRNAs into circulation [72].

Filarial infections are also common in humans and represent significant public health problems, especially in tropical areas. Besides *D. immitis*, other filarial parasites that usually infect mammals have been reported to occasionally infect humans, such as *D. repens*, *D. ursi*, and *D. tenuis* [74]. In Europe, the main concern is *D. repens* since its infections have increased drastically in the last decades and are now considered an emerging zoonotic disease [69,72]. MicroRNA expression in humans with dirofilariasis has not yet been described, probably due to its general low prevalence and relevance. On the other hand, there are some studies about circulating miRNAs in humans with other, more common, filarial infections, such as the ones caused by *Onchocerca volvulus* and *Brugia malayi* [75,76].

### 2.6. Heart Failure

Heart failure (HF) is a complex clinical syndrome common in small animal practice, characterised by the sustained inability of the heart to produce a stroke volume that meets perfusion’s tissues’ needs [77]. Furthermore, there is increased venous congestion and hydrostatic capillary pressure in the final stages of HF, favouring the development of interstitial oedema, described as CHF [78]. MicroRNAs could influence gene expression changes which are consistent with the pathophysiology of HF [34]. This makes miRNAs potential biomarkers as they may offer valuable information on the severity of the disease and risk stratification and guide the therapy plan [79].

Jung et al. found significant differences between the miRNA expression profiles in dogs with CHF secondary to MMVD and healthy controls. Of all the 326 miRNAs identified, four (miR-133, miR-1, cfa-let-7e, and miR-125a) were significantly upregulated, and four (miR-30c, miR-128, miR-142, and miR-423) were downregulated in dogs with CHF. These downregulated miRNAs were associated, for example, with cardiac hypertrophy and endothelial-to-mesenchymal transition, which makes them potential biomarkers for this disease in dogs [80].

In experimental HF induced by ventricular tachypacing, fibroblasts appear to have a more robust response to miRNAs than cardiomyocytes, especially fibroblasts from the left atrium. Along with a few other miRNAs, miR-21 is most likely an essential contributor to profibrotic collagen production. The atrial selective fibrotic reaction in HF cardiac remodelling is caused by numerous miRNA changes in response to a CHF-inducing stressor [34].

In human medicine, the prevalence of HF is also rising, probably due to the ageing of the population and success in treating cardiovascular diseases that frequently precede HF. Therefore, there is a constant search for better approaches to diagnose, manage, and assess patients’ prognoses with HF [79]. Most veterinary studies based their miRNAs search on human medicine studies. Not all the significant expression changes in human medicine are translated into veterinary medicine [48]. On the other hand, some studies evaluated these molecules on heart tissues and others on plasma. Some expression changes may be confined to the heart and not be detected in the plasma, which is a limitation when comparing studies [64]. 

Nevertheless, natriuretic peptides are human medicine’s gold standard biomarkers for HF diagnosis and prognosis. Although miRNAs improve diagnostic predictability and are helpful in HF prognosis, there is no sufficient evidence for their standalone use as biomarkers [79]. Additionally, the administration of extracellular miRNAs is thought to be a potential novel therapy for HF [81].

### 2.7. Coronary Artery Disease and Myocardial Infarction

In veterinary medicine, acute myocardial infarctions due to atherosclerotic obstruction of a main coronary artery are uncommon [82]. Usually, an underlying systemic or cardiac cause preceded a thromboembolic state, including infectious endocarditis, neoplasia, acute pancreatitis, or corticosteroid use. Occasionally, strokes have been associated with patent ductus arteriosus, HCM, and mitral insufficiency. Coronary artery disease (CAD) has rarely been diagnosed in dogs suffering from a myocardial infarction. Humans have a strong correlation between atherosclerosis and strokes [57,83]. Dogs with severe hypothyroidism can develop atherosclerosis of the coronary arteries, but it hardly leads to myocardial infarction [84]. Also, the narrowing of intramural coronary arteries, microscopic myocardial infarctions, and focal myocardial fibrosis appears to be related to MMVD, even though older dogs who do not have valvular disease still have these vascular changes. Eventually, CAD causes CHF due to decreased myocardial function, which can be fatal. Some causes of CAD, such as diabetes in humans, take more than ten years to manifest its effects, explaining why this disease is so uncommon in animals [83].

In humans, CAD is the most frequent cause of unexpected cardiac death in adults and the most significant cause of global morbidity and mortality [85]. MicroRNAs are potential biomarkers of CAD since this disease and its risk factors (e.g., abnormal lipid metabolism, inflammation) cause alterations in miRNAs expression profiles [86]. Of the dysregulated miRNAs, miR-1, miR133a, miR-133b, miR-208, and miR-499 appeared to be the most promising for diagnosing acute myocardial infarction. On the other hand, miR-126, miR-199a, miR-132, miR-140-3p, and miR-210 seemed more useful as prognostic biomarkers [85]. In people with ST-segment elevation myocardial infarction, miR-208a is the most promising biomarker. It allows an earlier diagnosis compared with the gold standard, cardiac troponin (cTn), since it can be detected within 2 hours of the onset of acute myocardial infarction. Besides, within 24 hours, miR-208a values decline to baseline, allowing for the detection of other minor cardiac events post-infarction [87]. Despite their potential, miRNAs are not yet used in clinical practice because of their cost, unstandardised methodologies, and extensive research inconsistencies [85]. 

### 2.8. Cardiac Toxicity

Doxorubicin (DOX) is an anthracycline chemotherapeutic agent used to treat many tumours. Its use is limited due to the cumulative dose-dependent cardiotoxicity, manifesting as irreversible degenerative DCM, in human and veterinary patients. Three miRNAs were reported to be differentially expressed in canine patients after administration of DOX: miR-107 and miR-146a were significantly downregulated, while miR-502 was significantly upregulated [88].

Similarly, in human paediatric patients, it was also demonstrated downregulation of mir-107 and mir-146a [89]. In human medicine, many miRNAs have been reported to participate in multiple pathological processes that target different protein mRNAs and damage heart cells by inducing apoptosis, mitochondrial dysfunction, excessive reactive oxygen species, and endoplasmic reticulum stress [90,91].

MicroRNAs are promising biomarkers for cardiotoxicity detection as they may help clinicians modify treatment or implement early cardioprotective strategies. These strategies aim to minimise irreversible cardiac damage by therapeutically manipulating dysregulated miRNAs since miRNAs modulate entire signalling pathways. However, many miRNAs modulated by anticancer treatments are also involved in cardiotoxicity.

The following miRNAs have been demonstrated to be implicated in CVDs and are also modulated by therapies for cancer [92]. The miR-200 family comprises miR-200a, miR-200b, miR-200c, miR-141 and miR-429. This miRNA family induces the epithelial–mesenchymal transition of tumour cells and is involved in cardiovascular homeostasis affected by cancer treatments [93]. Specifically, it has been demonstrated that DOX induces upregulation of miR-200c in cardiac mesenchymal progenitor cells [94]. Moreover, miR-200c and other family members are oxidative stress-induced miRNAs that promote downmodulation of ZEB1 and are implicated in endothelial dysfunction, causing apoptosis and senescence in endothelial cells [95]. The miR-34 family, which includes miR-34a, b, and c, is modulated by different anti-cancer treatments. MiR-34a was upregulated in the myocardium and plasma of DOX-treated rats and DOX-induced rat cardiomyocyte H9c2 cells [96]. The miR-29 family is formed by miR-29a, b, and c and is also modulated by anti-cancer treatments [97]. These miRNAs play a crucial role in cardiac remodelling after cardiomyocyte injury and are regulators of cardiac fibrosis [98]. The miR-30 family comprises miR-30a, miR-30b, miR-30c, miR-30d, and miR-30e. It has been demonstrated that high levels of miR-30 protect against DOX toxicity and correlate with the decrease of reactive oxygen species [99]. Other miRNAs such as miR-21, miR-1, miR-133a/b, miR-499, and miR 208a/b have been studied in different cancer treatments that induce cardiotoxicity [92]. Therefore, it is imperative to fully understand the mechanisms of DOX-induced cardiotoxicity to develop effective methods for detecting cytotoxicity and therapeutic applications [100].

### 2.9. Hypertensive Vascular Conditions

Systemic hypertension (SH) refers to the consistent increase in systemic blood pressure. Systemic hypertension can be due to an environmental stressor associated with a disease that increases blood pressure or can happen without any identifiable cause (idiopathic). Secondary SH is associated with conditions known to cause high blood pressure, such as chronic kidney disease, hyperadrenocorticism, diabetes mellitus, and pheochromocytoma [101,102]. The renin–angiotensin–aldosterone system is a crucial point in maintaining an adequate extravascular volume and blood pressure [103]. Several miRNAs regulate renin–angiotensin–aldosterone system genes. In addition, it is described that the miRNA expression is altered in hypertensive human patients [104]. MiR-136 was significantly downregulated in peripheral blood serum of hypertensive patients [105]. Some miRNAs, like miR-202, were overexpressed and might exert a protective role against hypertension [106]. Other miRNAs with high expression in hypertensive patients were miR-21, miR-126, miR-196a, and miR-451, whereas miR-181a, miR-638, and miR-663 were under-expressed [107]. Moreover, it was noted that miR-181a, miR-663, and miR-25 regulate the renin gene [107,108]. The studies published in veterinary medicine only included animal models, specifically hypertensive rat models [109]. 

Pulmonary hypertension (PH) is a severe condition defined by increased pulmonary vascular resistance and pulmonary artery pressure [110]. MiR-150 levels were significantly reduced in human patients with PH and samples harvested from the lungs of a rat model of PH [111]. Schlosser et al. stated that circulating miR-26a decreases in rat models of PH and human patients with PH [112]. Moreover, it was observed that miR-26a, miR-29c, miR-34b, and miR-451 were downregulated, and miR-21, miR-130a, miR-133b, miR-191, miR-204, and miR-208b were upregulated. These plasma miRNAs were thought to be candidates for diagnostic biomarkers of PH in humans [113]. However, there are several limitations to using miRNAs as reliable biomarkers of PH, such as the lack of consistency in methods and results and the need for more extensive longitudinal studies [114]. 

## 3. Future Directions, Limitations, and Clinical Perspective

As described in this review, the expression of several miRNAs is altered in different CVDs. Table 1 provides a summary of the regulatory role of some miRNAs in cardiovascular diseases in dogs and cats. MicroRNAs may be used to distinguish different diseases’ stages so the treatment plan can be even more individualised, help monitor disease progression, or evaluate its prognosis. Moreover, a door has opened for new potential treatments for cardiac disorders. Knowing how physiology changes from a molecular point of view, we can theoretically tackle these illnesses by interrupting their aberrant microRNA expression. There are two main approaches for employing miRNAs as therapeutic agents, the first consists of reinstating the downregulated miRNA, using miRNA mimics, and the latter is based on hindering the overexpressed miRNA, applying miRNA inhibitors (anti-miRs) [115]. MicroRNA mimics are formulated to replace or increase the number of beneficial miRNAs. These are processed similarly to endogenous miRNA and will lower the level of specific genes. Anti-miRs are synthetic RNAs whose goal is inhibiting miRNAs that are overexpressed in disease. This can be achieved by targeting miRNAs for degradation or by sequestering the miRNA so it can no longer attach to its targets. Several strategies target miRNAs, such as antagomiRs (cholesterol-conjugated anti-miRs), locked nucleic acids, and antisense oligonucleotides [116].

To date, several miRNA-targeted therapeutics have reached clinical development in human medicine, often using animal models for their development [117,118]. However, using miRNAs as biomarkers is far from becoming a reality in the veterinary clinical setting. The logistics involved in sample processing are precise, the cost is high, and there is a lack of standardisation in techniques [119]. Moreover, extensive prospective studies are missing, and a large amount of evidence comes from monocentric case-control studies. These limitations represent inspiring challenges for future research to move the miRNA into the veterinary clinical practice hopefully. 

## 4. Conclusions

The discovery of noncoding RNA has revolutionised gene expression knowledge. MicroRNA’s contribution to diverse physiological pathways in the cardiovascular field is evident and highly promising but is yet to be fully understood. Furthermore, miRNA’s high stability in biological samples and their presence in circulation mirroring the changes within cells imply a potential role as biomarkers.

The understanding the pathobiology of miRNAs is a fundamental point in developing new diagnostic and therapeutic strategies. Overall, it has been extensively demonstrated that miRNAs play a crucial part in several CVDs. To this matter, a joint effort to analyse results, standardise methodologies and move towards validating miRNAs as biomarkers will undoubtedly translate into miRNA as a valuable tool in the future clinical setting. 

## Figures and Tables

**Table 1:** Regulatory role of some miRNAs in cardiovascular diseases in dogs and cats. Abbreviations: AF, atrial fibrillation; CHF, congestive heart failure; CTGF, connective tissue growth factor; DCM, dilated cardiomyopathy; HF, heart failure; miRNAs, microRNAs; MMVD, myxomatous mitral valve disease.

miRNA	Change in Expression	Purpose	Sample	Comments	Reference
Atrial Fibrillation					
29b	↓	Diagnosis and therapeutic target	Cardiac tissue	MiR-29 likely plays a role in atrial fibrotic remodelling	[33]
21	↑	Diagnosis	Cardiac tissue	Several miRNA changes are essential to atrial-selective fibrotic response, but miR-21 is probably one of the most relevant contributors.	[34]
133	↓	Diagnosis	Cardiac tissue	MiR-133 and miR-30 are anti-fibrotic miRNAs and are differentially expressed in chronic AF.	[35]
30	↓				
132	↓	Therapeutic target	Cardiac tissue	MiR-132 expression decreases, and CTGF increases in humans and canine models with AF.	[36]
206	↑	Therapeutic target	Cardiac tissue	MiR-206 overexpression induces cardiac autonomic nerve remodelling and enables AF.	[38]
Myxomatous mitral valve disease					
9	↑	Disease monitoring and therapeutic target	Plasma	Exosomal miRNA expression level seems to be specific to disease states. Note that cfa-miR-495 decreased in total plasma but increased in plasma exosomes.	[45]
181c	↑				
495	↑				
599	↓				
let-7c	↓	Therapeutic target	Cardiac tissue	Epigenetic dysregulation probably participates in canine MMVD evolution	[46]
17	↓				
20a	↓				
30d	↓				
30b	↓	Diagnosis	Plasma	MiR-30b is downregulated when comparing dogs with MMVD and controls MiR-133b is downregulated in dogs with CHF secondary to MMVD	[48]
133b	↓				
302d	↓	Diagnosis, prognosis, or monitoring treatment	Serum	Dogs with stage B1/B2 or stage C/D compared to stage A	[50]
380	↓				
874	↓				
582	↓				
490	↓				
329b	↓				
487b	↓				
103	↑				
98	↑				
cfa-let-7b	↑				
cfa-let-7c	↑				
130b	↑	Diagnosis or therapeutic target	Serum	Dogs with stages C/D compared to stage A.	[55]
375	↓				
425	↓				
30d	↓				
30c	↓				
151	↓				
cfa-let-7b	↓				
19b	↓				
cfa-let-7g	↓				
cfa-miR-130b	↑	Diagnosis or therapeutic target	Serum	Dogs with stage B compared to healthy dogs.	[51]
Cardiomyopathies					
142-3p	↑	Diagnosis	Serum	Results from dogs with DCM. There was no statistical significance, probably due to the small sample size.	[58]
144*	↑				
21	-				
let-7c	↓				
92a	-				
381-3p	↑	Diagnosis	Serum	Cats with hypertrophic cardiomyopathy.	[64]
486-3p	↑				
4751	↑				
476c-3p	↑				
5700	↑				
513a-3p	↑				
320e	↑				
1246	↑				
Heartworm disease					
34	↑	Diagnosis	Plasma	These miRNAs are filarial derived that can be found in the host bloodstream. These miRNAs were also detected in samples from dogs infected with *Brugia pahangi.*	[72]
71	↑				
Heart Failure					
9	↑	Predict development	Plasma	Expression precedes CHF, which suggests that these miRNAs are involved in the initiating process of CHF.	[45]
599	↓				
495	↑	Monitor progression	Plasma	Expression was only high at the time of disease.	
181c	↑				
133	↑	Diagnosis	Plasma	Circulating microRNA expression patterns were distinct and are presumably molecular biomarkers of CHF.	[80]
1	↑				
cfa-let-7e	↑				
125a	↑				
30c	↓				
128	↓				
142	↓				
423	↓				
21	↑	Therapeutic target	Cardiac tissue	Results from experimental HF induced by ventricular tachypacing.	[34]
Cardiac Toxicity					
107	↓	Diagnosis	Plasma	Downregulation of miR-502 was detected before significant changes in cTnI concentrations or echocardiographic parameters.	[88]
146a	**↓**				
502	**↑**				
181d	**↑**				

↑ upregulation, ↓ downregulation.

## Data Availability

This paper did not report any data.
